# Short-term creatine supplementation enhances strength, reduces fatigue, and accelerates recovery in resistance-trained athletes: a double-blind, randomized, crossover trial

**DOI:** 10.1080/15502783.2026.2617283

**Published:** 2026-01-24

**Authors:** Atef Salem, Achraf Ammar, Mohamed Kerkeni, Mohamed Ali Boujelbane, Ayse Merve Uyar, Leonard Moritz Köbel, Saranya Selvaraj, Reza Zare, Katie M. Heinrich, Haitham Jahrami, Slim Tounsi, Giuseppe Grosso, Wolfgang I. Schöllhorn, Khaled Trabelsi, Hamdi Chtourou

**Affiliations:** a Department of Training and Movement Science, Institute of Sport Science, Johannes Gutenberg-University Mainz, Mainz, Germany; b High Institute of Sport and Physical Education of Sfax, University of Sfax, Sfax, Tunisia; c Research Laboratory, Molecular Bases of Human Pathology, LR19ES13, Faculty of Medicine of Sfax, University of Sfax, Sfax, Tunisia; d Research Laboratory: Education, Motricity, Sport and Health, EM2S, LR19JS01, High Institute of Sport and Physical Education of Sfax, University of Sfax, Sfax, Tunisia; e Department of Chemistry, Faculty of Applied Sciences, University of Sri Jayewardenepura, Gangodawila, Nugegoda, Sri Lanka; f SRH Campus Hamburg, SRH University of Applied Sciences Heidelberg, Hamburg, Germany; g Department of Kinesiology, Kansas State University, Manhattan, KS, USA; h Department of Research and Evaluation, The Phoenix, Denver, CO, USA; i Government Hospitals, Manama, Bahrain; j Department of Psychiatry, College of Medicine and Medical Sciences, Arabian Gulf University, Manama, Bahrain; k Laboratory of Biopesticides (LBPES), Center of Biotechnology of Sfax, University of Sfax, Sfax, Tunisia; l Department of Biomedical and Biotechnological Sciences, University of Catania, Catania, Italy; m Department of Movement Sciences and Sports Training, School of Sport Science, The University of Jordan, Amman, Jordan; n Research Unit, Physical Activity, Sport, and Health, UR18JS01, National Observatory of Sport, Tunis, Tunisia

**Keywords:** Creatine, ergogenic effect, resistance exercise, performance, recovery

## Abstract

**Background:**

While the long-term ergogenic benefits of creatine monohydrate (CrM) supplementation are well-documented, the potential advantages of acute ingestion followed by short-term consumption remain relatively underexplored. This double-blind, randomized crossover study investigated the acute and short-term effects of CrM supplementation on strength performance, heart rate variability (HRV) responses, and recovery of lower limbs strength and muscle soreness in resistance-trained males.

**Methods:**

A total of eleven physically active participants were recruited; however, due to incomplete data, one participant was excluded, and ten participants (age: 21.3 ± 1.9 years) were analyzed. Participants ingested either creatine monohydrate (CrM: 0.3 g·kg^−1^·d^−1^) or a placebo (PLA) for three days, with the first day’s dose consumed 2 h pre-test and subsequent doses divided into three daily doses. Participants completed two test sessions in a randomized order, separated by a seven-day washout period. Each session included bench press (BP) and back squat (BS) tests performed at 60%, 70%, and 80% of one-repetition maximum (1RM) under either CrM or PLA conditions. Strength performances (repetitions, velocity, power), HRV and peak heart rate (HR), jump tests (Countermovement Jump (CMJ) and Squat Jump (SJ)), and delayed-onset muscle soreness (DOMS) were assessed.

**Results:**

Compared to PLA, CrM supplementation significantly increased repetitions completed at 60−80% 1RM in BP and BS (*p* ≤ 0.041, *d* = 0.72–1.6) during both test sessions. CrM reported higher velocity compared to PLA at all intensities (60–80% 1RM) for both exercises and sessions (*p* ≤ 0.035, *d* = 0.78–4.09). CrM reduced cardiovascular strain compared to PLA at 60% 1RM during back squat (*p* = 0.017, *d* = 1.05). Peak HR increased with intensity for both conditions (*d* = 1.1–4.28), with CrM showing lower HR at 60% (*p* = 0.017, *d* = 1.05) and higher HR at 80% (*p* = 0.047, *d* = 0.82) compared to PLA. CrM enhanced post-exercise parasympathetic reactivation in the 1^st^ session demonstrating higher favorable response in RMSSD (*p* = 0.015, *d* = 2.99) and HF power (*p* = 0.022, *d* = 2.76) compared to PLA. CMJ performance was higher in CrM compared to PLA at 24 h post-1^st^ session and immediately before and after the 2^nd^ session (*p* ≤ 0.019, *d* = 1.10–1.93), also DOMS was reduced in upper and lower limbs (*p* ≤ 0.012, *d* = 1.15–1.04) immediately before the 2^nd^ session.

**Conclusion:**

These findings demonstrate that even three days of CrM supplementation have the potential to enhance strength performance, reduce physiological stress, and accelerate recovery, suggesting it as an effective ergogenic strategy for athletes seeking immediate performance gains and reduced post-exercise soreness.

## 1. Introduction

In the field of sports performance and exercise science, athletes and active individuals seek strategies to enhance performance, health, and physical capabilities [1,2]. Training, recovery, and nutrition play key roles, with dietary supplementation, particularly creatine monohydrate (CrM), standing out due to strong scientific support [3,4]. Creatine (CR), a nitrogenous amino acid in skeletal muscle, has been extensively studied since the 1990s for its ability to increase intramuscular phosphocreatine (PCr) stores [5] and improve exercise performance [6,7]. Over three decades, CrM has been found to be one of the most effective, safe, and well-researched ergogenic aids, benefiting both athletes and older adults [4,8].

CR’s efficacy stems from its role in cellular energy metabolism. As part of the adenosine triphosphate-phosphocreatine (ATP-PCr), or phosphagen system (PCr) rapidly regenerates ATP during high-intensity activities like weightlifting and sprinting [7,9]. By buffering ATP depletion and reducing adenosine diphosphate accumulation, CR supplementation sustains maximal effort, delays fatigue and improves recovery [10,11]. Beyond immediate performance gains, CR supports long-term adaptations, increasing lean muscle mass, strength, power output, anaerobic threshold, and work capacity [12,13]. With a strong safety profile and minimal adverse effects, CR remains widely accepted in both scientific and consumer circles [4]. However, most studies have been conducted predominantly in males, as fluctuations in sex hormones across the menstrual cycle, and their modulation by hormonal contraceptives, can influence creatine kinetics and related physiological responses [14–17]. In line with this precedent and to maximise internal validity, the present investigation enroled only males’ participants.

The evolution of CrM supplementation strategies reflect decades of research aimed at optimising its bioavailability and practicality. A loading phase of 20 g/day (divided into four 5 g doses) over five days was recommended [5], which remains the gold standard for rapidly saturating muscle CR reserves and obtaining a 20−40% increase in PCr levels [18]. Moreover, smaller maintenance doses (2–5 g/day) could sustain these elevated stores [19], while alternative approaches, such as prolonged lower-dose regimens (e.g. 3 g/day for 28 days), were shown to achieve similar saturation without a loading phase [20]. Accordingly, the loading protocol (20 g/day over 5 days) remains the fastest way to saturate intramuscular CR stores (≈5–7 days), which can advance the onset of ergogenic effects in tasks dependent on ATP-PCr turnover; by contrast, daily 3–5 g without loading typically achieves similar saturation over ~3–4 weeks. Thus, loading accelerates time-to-benefit rather than conferring a unique long-term advantage [21,22]. Notably, very short supplementation windows show mixed outcomes across exercise models (e.g. improvements after 5 days in some studies vs. null results in others) [23].

Recent work has re-examined abbreviated and lower-dose CR strategies, including relative dosing schemes, timing around exercise, and dosing patterns that may enhance retention (e.g. small, frequent intakes), while confirming that loading mainly shortens time-to-saturation rather than uniquely enhancing ultimate gains [4,21,22,24–26]. For example, a 5-day loading protocol has improved maximal strength and anaerobic power in trained athletes, whereas a 2-day loading protocol generally fails to elicit ergogenic effects, and some models report null results even after 5 days [23]. Distributing ~20 g/day into smaller, frequent intakes (e.g. 1 g every 30 min) reduces urinary CR loss and likely improves intramuscular retention [27]. While these findings improve dosing strategies, the benefits of acute ingestion followed by short-term use; an approach suited for athletes training multiple times per week; remain underexplored. Typical resistance-training guidelines prescribe training each major muscle group 2–3 times per week with ~48–72 h between sessions, which aligns with the schedules of most recreational and strength athletes [28,29]. A supplementation protocol that enhances strength while supporting recovery would be highly relevant to resistance-trained populations who train multiple times per week [4,30]. Beyond performance outcomes, many studies have evaluated recovery-related indices with CR (e.g. muscle damage and inflammation markers, soreness, and strength/performance restoration) with mixed results influenced by training status, dosing/timing, and the post-exercise time window [4,24,30]. HRV is a practical, non-invasive marker of autonomic balance that tracks training load, fatigue, and recovery in athletes, where decreases in vagal-derived indices generally reflect higher sympathetic strain, while increases indicate favourable adaptation or readiness to train [31]. However, relatively few studies have examined acute autonomic responses, assessed via HRV, during resistance exercise across repeated sessions in resistance-trained individuals. Whether CrM alters HRV—a sensitive indicator of autonomic balance and recovery in athletes [32] and non-athletic populations [33]—remains unclear. Mechanistically, CR’s augmentation of the ATP-PCr system may limit exercise-induced metabolite buildup, dampening group III/IV muscle afferent feedback (muscle metaboreflex) that elevates sympathetic outflow; consequently, short-term CR could plausibly modulate HRV during and after resistance exercise [34]. Empirically, CR has been associated with altered cardiovascular/thermoregulatory strain in some contexts, and studies assessing autonomic outcomes report mixed findings; including no change in HRV in some protocols and shifts in parasympathetic indices in others; underscoring the need to clarify responses specifically within resistance-training models [35,36].

Evidence is limited on whether an acute dose of CrM followed by short-term CrM supplementation (i) improves performance across repeated resistance-training sessions in trained males, (ii) alters HRV during and after lifting, and (iii) accelerates recovery of lower-limb strength and soreness. To address these specific gaps, this double-blind, randomised, crossover study investigated the effects of acute ingestion followed by short-term CrM supplementation on strength performance, HRV responses, and the recovery of lower limb strength and muscle soreness in resistance-trained males. We hypothesised that acute ingestion followed by short-term CrM supplementation would (i) enhance strength performance across successive training sessions, (ii) positively modulate HRV responses, indicating improved autonomic regulation, and (iii) accelerate recovery by preserving lower limb strength and reducing muscle soreness.

## 2. Methods

### 2.1. Population

A priori power analysis using G*Power (v. 3.1.5.1) determined that a minimum of nine participants was necessary. The calculation targeted our primary endpoint for total load lifted (kg) in back squat (BS) across sets, using a repeated-measures ANOVA (condition × intensity × time). We assumed a large effect size (Cohen’s f = 0.50; ≈ η_p_² ≈ 0.20 based on prior creatine performance data [37]), *α* = 0.05, power (1–β) = 0.95, and a within-subject correlation of 0.50, which indicated *n* = 9.

A total of eleven recreationally resistance-trained males were recruited; however, due to incomplete data, one participant was excluded, and a total of ten participants were analyzed (age: 21.3 ± 1.9 y [range: 19–24]; BMI: 21.42 ± 2.36 kg·m^−2^ [18.6–25.1]; 1RM BS: 97 ± 14.18 kg [80–120]; 1RM bench press (BP): 56.36 ± 4.52 kg [50–60]). Training experience was ~3 months on average (median 12 weeks, range 6–24 weeks). All participants had engaged in structured resistance training (≥3 times per week for ≥6 weeks) before the study and were familiar with BP and BS exercises. All were CR-supplementation naïve at baseline. Exclusion criteria included the use of medications/supplements affecting muscle biology (e.g. corticosteroids, CrM, anabolic/nutritional substances) during/one month pre-study; pre-existing kidney/liver conditions, low blood pressure, or injuries hindering exercise (confirmed via interviews). Non-compliant participants or those failing to complete all study stages were excluded from the analyses. Participants maintained regular diets and avoided strenuous activity/NSAIDs to limit muscle turnover confounders [38]. Participants abstained from strenuous exercise for 48 h before each testing visit and maintained usual activity otherwise. Before participation, individuals were informed of the study's risks, benefits, and objectives, and provided written consent. The study followed the Declaration of Helsinki and received ethical approval from the local Research Ethics Committee of High Institute of Sport and Physical Education of El Kef, El Kef, Tunisia on 12 October 2024 (ISSEPK-0033/2024). The study was registered at the Pan African Clinical Trials Registry database on 15 May 2025 (PACTR202505827886996). To eliminate confounding variables from hormonal fluctuations, this study's sample was limited to males. Female sex hormones can impact HRV, strength, CrM response, and exercise-induced muscle damage indicators [39]. Additionally, the logistical constraints of this pilot study precluded the inclusion of additional sessions necessary to control for the menstrual cycle.

### 2.2. Experimentation protocol

The study followed a double-blind, crossover, randomised controlled design. The experimental procedure included one familiarisation session and four testing sessions, each separated by seven days. This interval was selected pragmatically to minimise acute carryover and allow sufficient recovery between sessions while maintaining ecological validity within a short-term crossover design. Although full normalisation of intramuscular CR typically requires four to six weeks following cessation [4,24], several recent crossover studies in applied sport contexts have adopted similar seven-day washouts [40,41] as an acceptable compromise to preserve training continuity and participant adherence [10]. The seven-day spacing also supported optimal participant recovery [42]. All experimental tests were conducted in the afternoon to reduce diurnal variability and to standardise testing time for neuromuscular performance and HRV across. Afternoon testing was chosen based on evidence indicating superior strength performance, lower perceived exertion, and more stable physiological responses during these hours [43,44]. Testing sessions were carried out at a controlled temperature of 24 °C (±1 °C) and at a consistent time of day (±0.5 hours) for each participant to minimise the impact of circadian rhythm variations [43,45].

**Figure 1.**
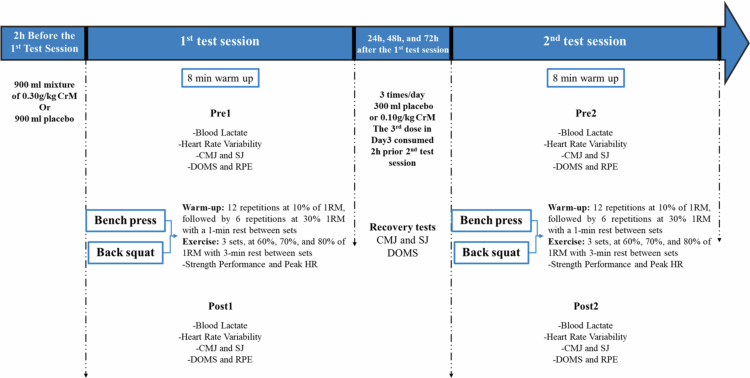
Experimental design. CrM: Creatine monohydrate; 1RM: One Repetition Maximum; Pre1: Before the 1st session; Post1: After the 1st session; Pre2: Before the 2nd session; Post2: After the 2nd session; HR: Heart rate; CMJ: Countermovement jump; SJ: Squat jump; DOMS: delayed onset of muscle soreness; RPE: Rating of perceived exertion.

During the first visit, participants’ anthropometric measurements were recorded using bioimpedance analysis (Tanita MC-780MA; Tanita Corporation, Japan). Additionally, they participated in a familiarisation session for the lifting protocol, emphasising maximum explosive velocity during both the BS and BP. A standardised warm-up was performed before determining their 1RM, following the ASEP guidelines for accurately assessing muscular strength and power [46]. The 1RM testing protocol was applied to both BS and BP within the same session. Participants first performed five repetitions at 50% of their estimated 1RM, followed by three repetitions at 70%, with 3-minute rest intervals between sets. After an additional 3-minute rest, participants attempted to reach their 1RM within five trials, each separated by a 3-minute rest period. Movement velocity was continuously tracked to confirm that the 1RM load corresponded to the expected speed for a true 1RM in each exercise [47,48]. The final 1RM values were then used to determine intensity percentages for the subsequent testing sessions. After familiarisation, participants returned to the laboratory four more times for each supplementation protocol (CrM and placebo (PLA)), performing an incremental resistance training test to assess the acute ergogenic effects of supplementation. In total, each participant completed four test sessions over a two-week period (Figure 1).

### 2.3. Supplementation protocol

In this study, CrM (GymBeam GmbH, Berlin, Germany) was mixed with placebo juice, ensuring identical colour, texture, and appearance for blinding. Two independent individuals handled randomisation and kit preparation, which included the assigned supplement, instructions, measuring spoons, and a water bottle. Researchers remained blinded until data collection ended. Blinding efficacy was assessed at the final visit, before debriefing. Participants guessed the supplement received in each phase and rated their confidence.

CrM was administered at 0.3 g·kg^−1^·d^−1^ for three days, a well-tolerated dosage for rapidly increasing muscle CR stores. On day one, the full dose was taken two hours before testing; on days two and three, it was divided into three sub-doses (0.1 g·kg^−1^·d^−1^). Supplements were provided in a shaker bottle with volume gradations. Participants refrained from food or drink (except water) two hours before testing and avoided other supplements on non-testing days. Adherence was tracked via a compliance log. Participants were instructed to: (i) avoid CR-rich foods, stimulants, gum, sweets, and alcohol for three days before testing; (ii) stay adequately hydrated; (iii) avoid strenuous exercise during the study; and (iv) sleep at least eight hours per night.

### 2.4. Strength exercise protocol

During each visit, participants completed an 8-minute supervised warm-up on a treadmill before performing BS and then BP on a Smith machine, with a 3-minute rest between exercises. Prior to the incremental strength test, they warmed up with 12 reps at 10% of 1RM and 6 reps at 30% of 1RM (with 1-minute rests) [49], followed by a 2-minute rest. The test consisted of three sets at 60%, 70%, and 80% of 1RM, performing repetitions to failure with 3-minute rests between sets [50]. Both the BS and BP were executed with a full range of motion—knee flexion and extension for the BS and elbow flexion and extension for the BP [51]. Participants were instructed to perform the concentric phase of each repetition at maximum velocity to optimise muscle strength development [48]. The entire testing session, including the warm-up, lasted approximately 35 minutes.

### 2.5. Measurements

#### 2.5.1. Incremental strength test and performance

Throughout the study, the total load lifted (in kg) and the maximum number of repetitions performed at 60%, 70%, and 80% of 1RM until failure were recorded for both the BS and BP. Additionally, Maximum velocity (MV) and power (MP) per set were tracked in real-time using the validated “Vmax Pro” accelerometer (high validity: R² ≥ 0.93 vs. Vicon/T-Force systems) during squats [52].

#### 2.5.2. Blood lactate measurement

Blood lactate levels were measured before, immediately after, and 3 minutes following the testing sessions [53]. These measurements were taken using the Lactate Pro 2 device Lactate Pro 2 (AKRAY Europe B.V. Prof J.H Bavincklaan51,183 AT, Amstelveen, the Netherlands) [54]. Samples were collected from the ear lobe, a standard sampling site [55], after the area was cleaned and sterilised with 70% ethanol.

#### 2.5.3. Heart rate variability (HRV) monitoring

HRV was monitored 5 minutes before, during, and after testing using a Polar H10 heart rate monitor with a Pro Strap, with data analysed via the Elite HRV app [56]. Time-domain parameters included Mean RR interval (MeanRR), root mean square of successive differences (RMSSD), and standard deviation of normal-to-normal intervals (SDNN), while frequency-domain analysis examined low-frequency (LF) and high-frequency (HF) components. Peak heart rate (HR) during exercise was also recorded.

#### 2.5.4. Countermovement jump (CMJ) and squat jump (SJ) tests

Jump performances were measured using the My Jump 2 app [57]. For the SJ, participants were asked to perform a maximal vertical jump with hands on the waist, starting from an angle of 90° at the knee. For the CMJ, the participants performed a maximal vertical jump starting from a standing position, with arm swing not allowed. Participants were given three trials for each test and the best trial was subsequently used for further analysis.

#### 2.5.5. Delayed Onset Muscle Soreness (DOMS)

DOMS was assessed at 24, 48, and 72 hours following the first test session for each supplementation protocol. Participants were asked to rate the degree of soreness in their knee extensors and elbow flexors using a visual analogue scale ranging from 0 to 10, where 0 represented no soreness and 10 indicated unbearable soreness [58,59].

### 2.6. Statistical analysis

Statistical analyses were conducted using the R programming language [60]. Descriptive statistics were presented as Mean ± standard deviation (SD). The visualization was conducted with “ggplot2” package [61]. We tested whether the proportion of correct treatment guesses differed from chance (50%) using a chi-square goodness-of-fit test with Yates’ continuity correction (df = 1). The normality of the data was checked using the Shapiro-Wilk test with the “rstatix” package [62]. To assess the statistical effects of supplementation condition, time, and intensity, a three-way repeated measures analysis of variance (ANOVA) with Greenhouse-Geisser correction was performed. When significant main or interaction effects were found, a post-hoc pairwise comparison with Bonferroni adjustment, was performed. The delta change (*∆pre-post*) was calculated as follows: *∆pre–post* (%) = ((score at post-session − score at pre-session)/score at pre-session) × 100. To assess the difference between supplementation condition and time effects of delta changes for HRV indices, a two-way ANOVA was performed, followed by a post-hoc pairwise comparison with the Bonferroni adjustment. All ANOVA models were conducted with the “afex” package [63] and the pairwise comparisons with “emmeans” package [64]. The effect size statistic was calculated as partial eta-squared (η²p) to assess the magnitude of the effects as small (0.01), moderate (0.06), and large (0.14) [65]. Standardised effect size (Cohen’s d) analysis was used to interpret the magnitude of differences between means and classified according to Hopkins [66] as: trivial (*d* ≤ 0.20), small (0.20 < *d* ≤ 0.60), moderate (0.60 < *d* ≤ 1.20), large (1.20 < *d* ≤ 2.0), very large (2.0 < *d* ≤ 4.0), and extremely large (*d* > 4.0). Significance was accepted for all analyses at the a priori level of *p* < 0.05.

## 3. Results

### 3.1. Dietary intake

No significant differences in energy or macronutrient intake (Table 1) were found between conditions (*p* > 0.05).

**Table 1.** Dietary intake 24 hours before sessions under CrM and PLA conditions (mean ± SD).

Dietary intake (Mean ± SD)	PLA	CrM
Energy (kcal/day)	2355 ± 288	2485 ± 305
Carbohydrates (g/day)	302.1 ± 38.6	318.6 ± 41.2
Protein (g/day)	108.7 ± 16.9	116.2 ± 18.7
Fat (g/day)	83.4 ± 11.3	89.3 ± 12.6

Blinding assessment indicated guess accuracy near chance. Overall, 55% of participants correctly guessed whether they had received CrM or PLA, which did not differ from chance (χ²(1) = 0.20, *p* = 0.65), supporting effective blinding.

### 3.2. Reached repetitions

**Figure 2.**
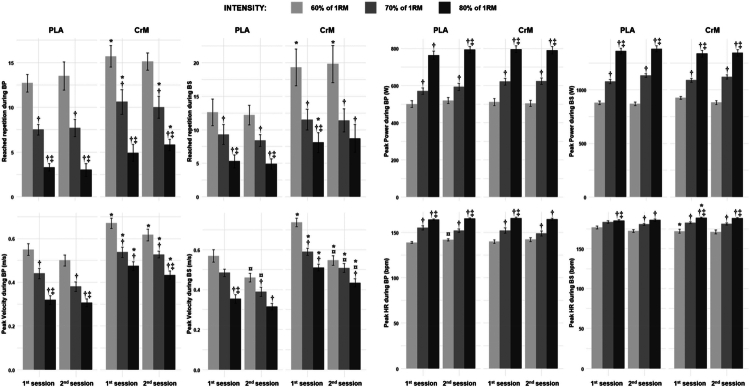
Reached repetition, peak velocity, peak power, and peak heart rate (HR) recorded during Bench press (BP) and Back squat (BS) incremental tests at two sessions for creatine monohydrate (CrM) and placebo (PLA) conditions. †: significantly different compared to 60% RM; ‡: significantly different compared to 70% RM; *: significantly different compared to PLA; ¤: significantly different compared to 1st session.

During the BP, there were significant main effects of condition and intensity (Figure 2, Table 2). Significant differences in reached repetition between all intensities across conditions and sessions were observed (*p* < 0.01, *d* = 1.52−4.2). Repetitions were significantly higher in the CrM compared to PLA condition at 60% (*p* = 0.0034, *d* = 1.25) and 70% 1RM (*p* = 0.041, *d* = 0.75) during the 1^st^ session, and at 70% (*p* = 0.008, *d* = 1.6) and 80% 1RM (*p* = 0.002, *d* = 1.37) during the 2^nd^ session.

**Table 2.** Three-way RMANOVA results for reached repetitions, peak velocity, peak power and peak HR during bench press and back squat.

	Repetitions		Peak velocity		Peak power		Peak HR	
	BP	BS	BP	BS	BP	BS	BP	BS
C	***F(1, 9) = 32.38***	***F(1, 9) = 9.58***	***F(1, 9) = 53.17***	***F(1, 9) = 115.01***	***F(1, 9) = 9.27***	F(1, 9) = 0.01,	F(1, 9) = 0.67	F(1, 9) = 0.01
	****p* < 0.001***	****p* = 0.013***	****p* < 0.001***	****p* < 0.001***	****p* = 0.014***	*p* = 0.929	*p* = 0.435	*p* = 0.915
	***η²*p* = 0.782***	***η²*p* = 0.516***	***η²*p* = 0.855***	***η²*p* = 0.927***	***η²*p* = 0.507***	η²*p* = 0	η²*p* = 0.069	η²*p* = 0.001
S	F(1, 9) = 0.02	F(1, 9) = 0.04	***F(1, 9) = 5.67***	***F(1, 9) = 108.31***	F(1, 9) = 0.71	F(1, 9) = 0.66	F(1, 9) = 0.04	F(1, 9) = 2.07
	*p* = 0.885	*p* = 0.844	****p* = 0.041***	****p* < 0.001***	*p* = 0.420	*p* = 0.439	*p* = 0.840	*p* = 0.184
	η²*p* = 0.002	η²*p* = 0.005	***η²*p* = 0.387***	***η²*p* = 0.923***	η²*p* = 0.074	η²*p* = .068	η²*p* = 0.005	η²*p* = 0.187
I	***F(1.87, 16.87) = 202.64***	***F(1.65, 14.81) = 90.70***	***F(1.53, 13.80) = 116.84***	***F(1.53, 13.74) = 69.37***	***F(1.81, 16.26) = 228.94***	***F(1.55, 13.99) = 533.34***	***F(1.67, 15.01) = 298.55***	***F(1.43, 12.91) = 48.84***
	****p* < 0.001***	****p* < 0.001***	****p* < 0.001***	****p* < 0.001***	****p* < 0.001***	****p* < 0.001***	****p* < 0.001***	****p* < 0.001***
	***η²*p* = 0.957***	***η²*p* = 0.910***	***η²*p* = 0.928***	***η²*p* = 0.885***	***η²*p* = 0.962***	***η²*p* = 0.983***	***η²*p* = 0.971***	***η²*p* = 0.844***
C × S	F(1, 9) = 0.13	F(1, 9) = 0.20	F(1, 9) = 0.04	F(1, 9) = 1.73	F(1, 9) = 1.29	F(1, 9) = 0.90, *p* =	F(1, 9) = 0.18	F(1, 9) = 0.35
	*p* = 0.729	*p* = 0.666	*p* = 0.850	*p* = 0.221	*p* = 0.286	0.369	*p* = 0.683	*p* = 0.571
	η²*p* = 0.014	η²*p* = 0.022	η²*p* = 0.004	η²*p* = 0.161	η²*p* = 0.125	η²*p* = 0.091	η²*p* = 0.019	η²*p* = 0.037
C × I	F(1.68, 15.13) = 0.11	***F(1.71, 15.39) = 6.30***	F(1.45, 13.08) = 0.31	F(1.62, 14.56) = 0.33	F(1.47, 13.23) = 1.18	F(1.88, 16.93) = 1.84	F(1.61, 14.53) = 1.58	***F(1.98, 17.78) = 3.90***
	*p* = 0.867	****p* = 0.013***	*p* = 0.670	*p* = 0.680	*p* = 0.322	*p* = 0.190	*p* = 0.238	****p* = 0.040***
	η²*p* = 0.012	***η²*p* = 0.412***	η²*p* = 0.033	η²*p* = 0.035	η²*p* = 0.116	η²*p* = 0.170	η²*p* = 0.149	***η²*p* = 0.302***
S × I	F(1.35, 12.17) = 0.24	F(1.21, 10.89) = 0.18	F(1.65, 14.86) = 0.39	***F(2.00, 17.97) = 4.81***	F(1.88, 16.96) = 0.05	F(1.25, 11.28) = 2.69	F(1.33, 11.95) = 1.72	F(1.55, 13.91) = 1.28
	*p* = 0.701	*p* = 0.728	*p* = 0.644	****p* = 0.021***	*p* = 0.948	*p* = 0.124	*p* = 0.219	*p* = 0.300
	η²*p* = 0.026	η²*p* = 0.019	η²*p* = 0.042	***η²*p* = 0.348***	η²*p* = 0.005	η²*p* = 0.230	η²*p* = 0.161	η²*p* = 0.124
C × S × I	F(1.74, 15.63) = 1.86	F(1.66, 14.90) = 0.01	F(1.59, 14.28) = 0.85	F(1.85, 16.64) = 1.22	F(1.41, 12.70) = 0.04	F(1.76, 15.83) = 0.04	F(1.49, 13.42) = 0.08	F(1.70, 15.26) = 0.88
	*p* = 0.190	*p* = 0.987	*p* = 0.422	*p* = 0.316	*p* = 0.913	*p* = 0.941	*p* = 0.867	*p* = 0.418
	η²*p* = 0.172	η²*p* = 0	η²*p* = 0.087	η²*p* = 0.120	η²*p* = 0.004	η²*p* = 0.005	η²*p* = 0.009	η²*p* = 0.089

BP: Bench press; BS: Back Squat; C: Condition; S: Session; I: Intensity.

Concerning repetitions during the BS (Figure 2), there were significant main effects of condition and intensity, as well as a significant condition × intensity interaction (Table 2). Moreover, there were significant differences between all intensities within both conditions at all session (*p* < 0.05, *d* = 1.1−3.12), except between 70 and 80% 1RM for the CrM condition during the 2^nd^ session (*p* = 0.159). Additionally, the CrM condition led to significantly greater repetitions compared to PLA for 60% and 80% 1 RM at the 1^st^ (*p* = 0.036 and 0.016, *d* = 0.79 and 0.9, respectively) and only for 60% at the 2^nd^ session (*p* = 0.013, *d* = 0.97).

### 3.3. Peak velocity

The analysis revealed significant main effects of condition, session, and intensity (Figure 2, Table 2). Post-hoc comparisons revealed significant differences between all intensities across conditions and sessions (*p* < 0.05, *d* = 1.15−2.15), except for 70% vs 80% for CrMduring the 1^st^ session (*p* = 0.2228). Velocity during both sessions was significantly higher for the CrM condition as compared to PLA at 60% (1^st^: *p* = 0.007, *d* = 1.11; 2^nd^: *p* = 0.034, *d* = 0.78), 70% (1^st^: *p* = 0.024, *d* = 0.86; 2^nd^: *p* < 0.001, d = 2.95), and 80% 1RM (1^st^ session: *p* < 0.001, *d* = 2.91; 2^nd^: *p* < 0.001, *d* = 3.98).

During the BS (Figure 2), there were significant main effects of condition, session, and intensity, as well as a significant session × intensity interaction (Table 2). Post-hoc comparisons revealed a significant decrease for the CrM condition from 60% to 70% (*p* = 0.002, *d* = 1.6) during the 1^st^ session and from 60% to 80% 1RM during both sessions (*p* < 0.001 and 0.046, *d* = 3.61 and 0.95, respectively for the 1^st^ and 2^nd^ sessions). Additionally, the PLA condition’s velocity decreased significantly from 60% to 80% 1RM and from 70% to 80% 1RM during the 1^st^ session (*p* < 0.001 and 0.011, *d* = 2.25 and 1.27, respectively), and from 60% to 80% 1RM during the 2^nd^ session (*p* = 0.001, *d* = 1.79). In the PLA condition, velocity during the 1^st^ session was significantly higher than the 2^nd^ session at 60% (*p* = 0.033, *d* = 0.75) and 70% 1RM (*p* = 0.015, *d* = 0.66), In contrast, the CrM condition showed a significantly higher velocity during the 1^st^ session compared to 2^nd^ session at 60% (*p* < 0.001, *d* = 1.42), 70% (*p* = 0.006, *d* = 1.09), and 80% 1RM (*p* = 0.002, *d* = 1.46). During both sessions, the CrM condition was significantly greater than PLA at 60% (1^st^: *p* = 0.001, *d* = 2.83; 2^nd^: *p* = 0.035, *d* = 1.57), 70% (1^st^: *p* = 0.002, *d* = 2.66; 2^nd^: *p* = 0.018, *d* = 1.94), and 80% 1RM (1^st^: *p* < 0.001, *d* = 3.84; 2^nd^: *p* < 0.001, *d* = 4.09).

### 3.4. Peak power

The analysis revealed significant main effects of condition and intensity (Figure 2, Table 2). The power significantly increased across all intensities for both conditions during both sessions (*p* < 0.05, *d* = 1.16−4.71), except from 60% to 70% 1RM for the PLA condition during the 1^st^ session (*p* = 0.094).

Regarding power during the BS (Figure 2), there was only a significant main effect of intensity (Table 2), where power significantly increased across all intensities for both conditions during both sessions (*p* < 0.005, *d* = 1.98−4.97).

### 3.5. Peak HR

Analysis of peak HR during the BP revealed a significant main effect of intensity (Figure 2, Table 2). Peak HR showed significant increases across all intensities for both conditions during both sessions (*p* < 0.05, *d* = 1.1−4.28), but not from 60% to 70% 1RM for the CrM condition during the 2^nd^ session (*p* = 0.317). Additionally, peak HR during the 1^st^ session was significantly lower compared to the 2^nd^ session for the PLA condition at 60% RM (*p* = 0.03, *d* = 0.82).

During the BS, there was significant main effect of intensity, as well as a significant condition × intensity interaction (Figure 2, Table 2). Peak HR significantly increased across all intensities for both conditions during both sessions (*p* < 0.05, *d* = 1.32−3.57), but not for the PLA condition from 60% to 70% 1RM during the 1^st^ session (*p* = 0.105) or from 70% to 80% 1RM during the 2^nd^ session (*p* = 0.64). During the 1^st^ session, the CrM condition had significantly lower HR at 60% (*p* = 0.017, *d* = 1.05) and higher HR at 80% 1RM (*p* = 0.047, *d* = 0.82) compared to PLA.

### 3.6. ∆Pre-Post change in HRV indices and lactate

**Figure 3.**
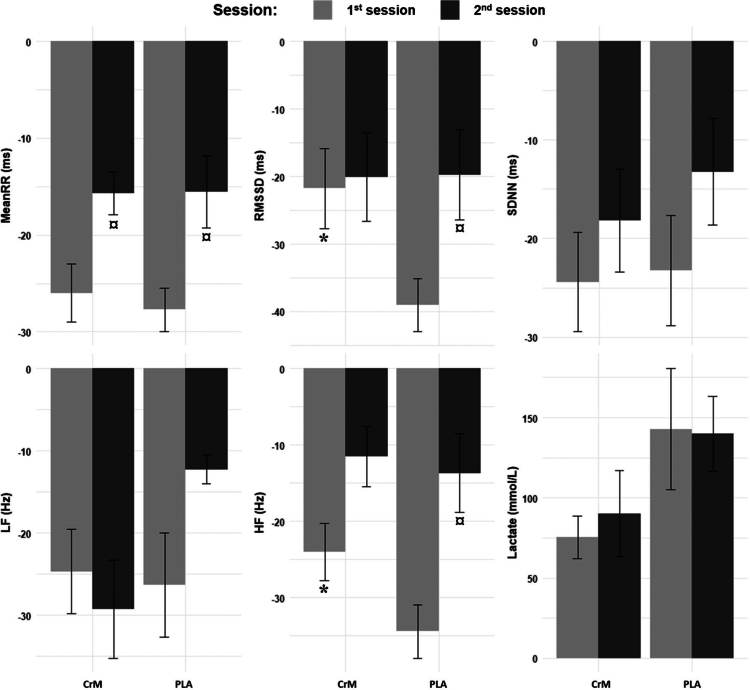
∆Pre-Post change in heart rate variability (HRV) indices and lactate for creatine monohydrate (CrM) and placebo (PLA) conditions. MeanRR: Mean RR interval; RMSSD: root mean square of successive differences; SDNN: standard deviation of normal-to-normal intervals; LF: low-frequency; HF: high-frequency (HF); *: significantly different compared to PLA; ¤: significantly different compared to 1st session.

Figure 3 represents the ∆Pre–Post changes (mean ± SD) for HRV indices and blood lactate for CrM and PLA conditions.

#### 3.6.1. Mean RR interval

The analysis for ∆pre-post change of meanRR revealed a significant main effect of session (F(1, 9) = 19.22, *p* = 0.002, η²*p* = 0.68), where ∆pre-post significantly decreased from the 1^st^ to the 2^nd^ session for both the PLA (*p* = 0.008, *d* = 3.37) and CrM conditions (*p* = 0.033, *d* = 2.5). However, no significant effects were found for condition (F(1, 9) = 0.05, *p* = 0.821, η²*p* = 0.006) or the condition × session interaction (F(1, 9) = 0.10, *p* = 0.755, η²*p* = 0.011).

#### 3.6.2. RMSSD

There was a significant main effect of session for RMSSD’s ∆pre-post change (F(1, 9) = 5.29, *p* = 0.047, η²*p* = 0.37), where ∆pre-post significantly decreased from the 1^st^ to the 2^nd^ session in the PLA condition (*p* = 0.006, *d* = 3.57). Additionally, the CrM condition had significantly higher ∆pre-post compared to PLA in the 1^st^ session (*p* = 0.015, *d* = 2.99). Nevertheless, no significant effects were found for condition (F(1, 9) = 2.42, *p* = 0.154, η²*p* = 0.21) or the condition × session interaction (F(1, 9) = 3.07, *p* = 0.114, η²*p* = 0.25).

#### 3.6.3. SDNN

There were no significant main effects for session (F(1, 9) = 2.93, *p* = 0.121, η²*p* = 0.25) or condition (F(1, 9) = 0.41, *p* = 0.539, η²*p* = 0.04), or condition × session interaction (F(1, 9) = 0.15, *p* = 0.710, η²*p* = 0.02) for SDNN.

#### 3.6.4. LF and HF

The analysis of LF’s ∆pre-post change revealed non-significant main effects of condition (F(1, 9) = 4.23, *p* = 0.070, η²*p* = 0.32), session (F(1, 9) = 0.42, *p* = 0.534, η²*p* = 0.04), or condition × session interaction (F(1, 9) = 3.37, *p* = 0.100, η²*p* = 0.27).

For HF power, a significant main effect of session was found (F(1, 9) = 22.90, *p* < 0.001, η²*p* = 0.72), where ∆pre-post of HF significantly decreased from the 1^st^ to the 2^nd^ session for the PLA condition (*p* = 0.003, *d* = 4.00). Additionally, there was a significant low pre-post change for the CrM condition in the 1^st^ session compared to PLA (*p* = 0.022, *d* = 2.76). Otherwise, no significant condition (F(1, 9) = 2.31, *p* = 0.163, η²*p* = 0.20) or condition × session interaction (F(1, 9) = 0.94, *p* = 0.358, η²*p* = 0.10) effects were found.

#### 3.6.5. Lactate

Data for ∆pre-post change of lactate showed no significant main effects of condition (F(1, 9) = 4.30, *p* = 0.068, η²*p* = 0.32), session (F(1, 9) = 0.05, *p* = 0.835, η²*p* = 0.005), or condition × session interaction (F(1, 9) = 0.12, *p* = 0.734, η²*p* = 0.014).

### 3.7. Post-session recovery

**Figure 4.**
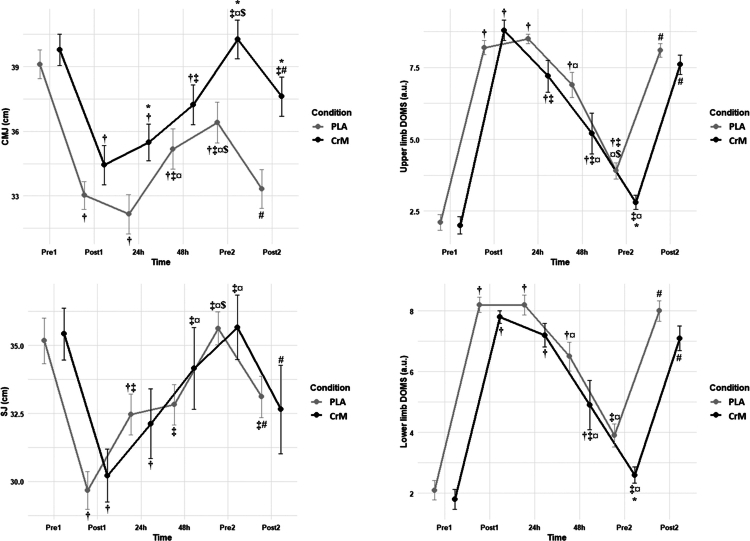
Countermovement jump (CMJ), squat jump (SJ), and delayed onset of muscle damage (DOMS) recorded pre- and post-1st sessions, 24 h, 48 h, and pre- and post-2nd session for for creatine monohydrate (CrM) and placebo (PLA) conditions. †: significantly different compared to Pre1; ‡: significantly different compared to Post2; ¤: significantly different compared to 24 h; $: significantly different compared to 48 h; #: significantly different compared to Pre2; *: significantly different compared to PLA.

Figure 4 represents the mean (±SD) changes in post-session recovery for CMJ, SJ, and upper and lower limb DOMS over time for CrM and PLA.

#### 3.7.1. CMJ

The analysis for CMJ revealed significant main effects of condition (F(1, 9) = 10.34, *p* = 0.011, η²*p* = 0.535), time (F(2.84, 25.53) = 150.73, *p* < 0.001, η²*p* = 0.944), and condition × time interaction (F(2.62, 23.62) = 9.98, *p* < 0.001, η²*p* = 0.526). In the PLA condition, significant decreases were observed from Pre1 to Post1 (*p* < 0.001, *d* = 5.29). Additionally, performance for the PLA condition at Pre1 was significantly higher compared to 24 h (*p* < 0.001, *d* = 5.06), 48 h (*p* = 0.001, *d* = 2.88), and Pre2 (*p* = 0.01, *d* = 1.61). A significant increase was found for the PLA condition from Post1 to 48 h (*p* = 0.016, *d* = 1.50) and Pre2 (*p* = 0.003, *d* = 1.95), from 24 h to 48 h (*p* < 0.001, *d* = 8.72) and Pre2 (*p* < 0.001, *d* = 5.67), and from 48 h to Pre2 (*p* = 0.006, *d* = 1.93). Similarly, for the CrM condition, significant decreases were observed from Pre1 to Post1 (*p* < 0.0001, *d* = 3.75). Moreover, CMJ for the CrM condition at Pre1 was significantly higher compared to 24 h (*p* < 0.001, *d* = 4.97) and 48 h (*p* = 0.012, *d* = 1.57). In contrast, significant increases were found for the CrM condition from Post1 to 48 h (*p* < 0.0001, *d* = −3.96), Pre2 (*p* < 0.001, *d* = 6.24), and from 24 h to Pre2 (*p* < 0.001, *d* = 3.14), and from 48 h to Pre2 (*p* < 0.001, *d* = 4.58). Additionally, a significant decrease was reported from Pre2 to Post2 (*p* = 0.001, *d* = 2.21). Moreover, the CrM condition was significantly higher in CMJ compared to PLA at 24 h (*p* = 0.006, *d* = 1.10), Pre2 (*p* = 0.003, *d* = 1.26), and Post2 (*p* = 0.002, *d* = 1.37).

#### 3.7.2. SJ

The analysis for SJ revealed a non-significant main effect of condition (F(1,9) = 0.04, *p* = 0.845, η²*p* = 0.004) and a non-significant condition × time interaction (F(2.13, 19.20) = 1.32, *p* = 0.292, η²*p* = 0.128). In the PLA condition, significant decreases were observed from Pre1 to Post1 (*p* < 0.001, *d* = 4.11). As well, SJ performance for the PLA condition was higher at Pre1 compared to 24 h (*p* = 0.008, *d* = 1.64). In contrast, significant increases were found for the PLA condition from Post1 to 24 h (*p* = 0.004, *d* = 1.93), 48 h (*p* = 0.005, *d* = 1.84), Pre2 (*p* < 0.001, *d* = 4.45), and Post2 (*p* = 0.011, *d* = 1.90), from 24 h to Pre2 (*p* = 0.005, *d* = 1.87), and from 48 h to Pre2 (*p* < 0.001, *d* = 4.46). Additionally, a significant decrease was found from Pre2 to Post2 (*p* < 0.001, *d* = 2.90). Regarding SJ performance for the CrM condition, significant decreases were observed from Pre1 to Post1 (*p* < 0.0001, *d* = 4.11) with Pre1 significantly higher than 24 h (*p* = 0.0059, *d* = 1.72). Significant increases were found from Post1 to 48 h (*p* = 0.0021, *d* = −2.10), Pre2 (*p* < 0.0001, *d* = −4.09) and from 24 h to 48 h (*p* = 0.0024, *d* = −2.07) and Pre2 (*p* = 0.0013, *d* = −2.38). Similarly, a significant decrease was observed from Pre2 to Post2 (*p* = 0.0199, *d* = 1.93) for the CrM condition. However, there was a significant main effect of time (F(2.34, 21.08) = 60.90, *p* < 0.001, η²*p* = 0.871).

#### 3.7.3. Upper limb DOMS

The analysis for the upper limb DOMS revealed a significant main effect of condition (F(1, 9) = 5.13, *p* = 0.050, η²*p* = 0.363) and time (F(2.60, 23.36) = 143.21, *p* < 0.001, η²*p* = 0.941), but not for the condition × time interaction (F(2.62, 23.57) = 2.67, *p* = 0.078, η²*p* = 0.229). Pairwise comparisons for PLA were revealed significant increases in upper limb DOMS from Pre1 to Post1 (*p* < 0.001, *d* = 5.11), and DOMS was significantly lower at Pre1 compared to 24 h (*p* < 0.001, *d* = 5.13), 48 h (*p* < 0.001, *d* = 3.25), and Pre2 (*p* = 0.0056, *d* = −6.35), and at Post1 compared to Pre2 (*p* < 0.001, *d* = 4.12). In contrast, a significant decrease was reported from 24 h to 48 h (*p* = 0.047, *d* = 2.31) and Pre2 (*p* < 0.001, *d* = 4.22). Additionally, upper limb DOMS significantly increased for the PLA condition from Pre2 to Post2 (*p* < 0.001, *d* = 5.77). For the CrM condition, a significant increase was observed in DOMS from Pre1 to Post1 (*p* < 0.001, *d* = 5.34). As well, upper limb DOMS was higher at Pre1 compared to 24 h (*p* < 0.001, *d* = −3.48) and to Post2 (*p* < 0.001, *d* = 4.10). In addition, a significant increase was revealed for the CrM condition from Post1 to 24 h (*p* = 0.047, *d* = 2.42) and to 48 h (*p* = 0.003, *d* = 3). Also, there were significant decreases from 24 h to 48 h (*p* = 0.047, *d* = 2.3) and Pre2 (*p* = 0.001, *d* = 3.14), and from Pre2 to Post2 (*p* < .001, *d* = 4.29). At Pre2, upper limb DOMS for the CrM condition was significantly higher compared to PLA (*p* = 0.012, *d* = 1.15).

#### 3.7.4. Lower limb DOMS

The analysis for lower limb DOMS revealed a significant main effect of condition (F(1, 9) = 12.93, *p* = 0.006, η²*p* ​ = 0.59) and time (F(2.42, 21.77) = 86.08, *p* < 0.001, η²*p* = 0.905, but not for the condition × time interaction (F(1.77, 15.93) = 0.96, *p* = 0.394, η²*p* = 0.1). For PLA, significant increases in lower limb DOMS were observed from Pre1 to Post1 (*p* < 0.001, *d* = 5.6), also to 24 h (*p* < 0.001, *d* = 8.28) and 48 h (*p* < 0.001, *d* = 3.26). There was also a significant decrease in lower limb DOMS from Post1 and Pre2 (*p* < 0.001, *d* = 3.22), as well as from 24 h to Pre2 (*p* < 0.001, *d* = 2.63). Additionally, DOMS increased from Pre2 to Post2 (*p* < 0.001, *d* = 2.38) in the PLA condition. For the CrM condition, significant decreases in lower limb DOMS were observed from Pre1 to Post1 (*p* < 0.0001, *d* = 7.35), 24 h (*p* < 0.001, *d* = 5.01), and to 48 h (*p* = 0.008, *d* = 1.76). There was a significant increase in lower limb DOMS from Post1 to 48 h (*p* = 0.046, *d* = 1.27), and from 24 h to 48 h (*p* = 0.031, *d* = 0.56) and Pre2 (*p* < 0.001, *d* = 3.41). Also, lower limb DOMS increased from Pre2 to Post2 in the CrM condition (*p* < 0.001, *d* = 2.3). Moreover, the CrM condition had significantly lower DOMS at Pre2 than PLA (*p* = 0.01, *d* = 1.04).

## 4. Discussion

This study aimed to examine the effectiveness of short-term CrM supplementation on strength performance, HRV, and post-exercise recovery of lower limb strength and DOMS. During incremental BP and BS exercises, CrM supplementation significantly enhanced performance compared to PLA. In the BP, participants taking CrM completed more repetitions at 60% and 70% of 1RM in the 1^st^ session, with even greater improvements at 70% and 80% 1RM in the 2^nd^ session. Similarly, during the BS, CrM supplementation led to more repetitions at both moderate (60% 1RM) and high intensities (80% 1RM) across both sessions. CrM also improved peak velocity in both exercises. In the BP, velocity increased at all intensities, while in the BS, velocity gains were observed consistently across both sessions. Peak power output followed a similar pattern, with BP showing increased power at all intensities, while BS power improvements were more pronounced at higher intensities. In addition to strength benefits, CrM supplementation reduced cardiovascular strain. Peak HR was lower during BS at both 60% and 80% 1RM in the 1^st^ session. Moreover, CrM supplementation showed increases in HRV indices such as RMSSD and HF power during the 1^st^ session. CrMsupplementation also contributed to better neuromuscular recovery. Additionally, CMJ performance, in both the CrM and PLA conditions, decreased immediately after 1^st^ session, increased at 24 h, 48 h, and prior to the 2^nd^ session, and decreased again after the 2^nd^ session. Additionally, CrM supplementation reduced muscle soreness compared to the PLA condition, with lower DOMS reported in both upper and lower limbs before the 2^nd^ session.

### 4.1. Acute vs. short-term effects of CrM supplementation

The ergogenic effects of CrM supplementation manifest distinctly across acute (single-session) and short-term (multi-session) periods. Acutely, CrM supplementation immediately enhanced strength performance, as evidenced by increased repetitions, velocity, and power during the first session. For BP, CrMelevated repetitions at 60% 1RM (*d* = 1.25) and 70% 1RM (*d* = 0.75), with peak velocity improvements across all intensities (60% 1RM: *d* = 1.11; 70% 1RM: *d* = 0.86; 80% 1RM: *d* = 2.91). Similarly, in BS, acute effects were observed at 60% (*d* = 0.79) and 80% 1RM (*d* = 0.9) for repetitions and at 60% (*d* = 2.83) and 70% 1RM (*d* = 2.66) for velocity. Peak power during BP rose acutely (*d* = 1.16–4.71), with maximal gains at 80% 1RM (*d* = 4.71). These findings align with CR’s rapid elevation of intramuscular PCr stores, which accelerates ATP resynthesis during near-maximal efforts [4]. Acute benefits may also stem from CR’s osmotic properties, which increase muscle water content and sarcoplasmic calcium handling, facilitating cross-bridge cycling [18,67].

Acute supplementation rapidly increases intramuscular CR content, as demonstrated by Harris, Söderlund [5], who reported that 20 g/day of CR for five days increases total CR storage by roughly 50%, with 20−40% stored as PCr. This biochemical adaptation translates directly to improved performance outcomes, where five days of CR loading (20 g/day) enhanced peak torque production during repeated maximal muscle contractions, particularly in later exercise bouts (bouts 2–4), compared to placebo [7]. Similarly, six days of CR supplementation (5 g/day) mitigated the decline in work output during ten consecutive six-second cycling sprints, reinforcing CR’s role in sustaining high-intensity performance [24]. These findings are in line with Birch, Noble [68], who reported significant increases in peak power (+4.5%) and total work during the first two of three 30-second cycling bouts following five-day CR loading (20 g/day). Notably, performance benefits diminished in the third bout, likely due to ATP depletion.

Mechanistically, acute CR supplementation preserves ATP availability, where five days of CR loading (20 g/day) increased intramuscular PCr by 15%, reducing ATP loss by 30% during repeated 30-second cycling sprints while facilitating a 4% increase in total work output [69]. This highlights CR’s effectiveness in buffering energy demands during near-maximal efforts, reported single 20 g CR doses to improved cycling sprint power due to rapid PCr saturation [70].

When acute ingestion was followed by short-term supplementation (up to three days of CrM intake), the ergogenic effects were further amplified in the subsequent training session, particularly at higher intensities (70–80% 1RM). The second session demonstrated greater BP repetitions at 80% 1RM (*d* = 1.37) and increased BS peak velocity (*d* = 4.09), suggesting cumulative performance benefits from repeated CrM dosing. This aligns with Candow, Forbes [71], who observed that five days of CrM loading (20 g/day) increased intramuscular CR retention by ~15%, subsequently enhancing proton buffering during repeated sprint efforts. The intensity-dependent power gains in BP (*d* = 4.71 at 80% 1RM) and BS (*d* = 4.97 at 80% 1RM) underscore CR’s critical role in maintaining ATP availability [24]. Interestingly, HR did not significantly decrease during the second session, contrasting with acute HRV improvements, indicating that residual fatigue might obscure cardiovascular benefits during repeated bouts [72].

Overall, acute performance enhancements may result from CrM’s rapid PCr saturation, which buffers ATP depletion during high-intensity efforts [6]. Short-term use could also upregulate the mammalian target of rapamycin (mTOR) signalling and glycogen storage [73], while CR’s antioxidant properties may help mitigate oxidative damage [74]. Additionally, emerging evidence suggests that CrM may play a role in satellite cell activation, potentially facilitating muscle repair [67]. Short-term supplementation protocols (up to six weeks) further amplify these benefits by sustaining intramuscular CR retention. Van Loon, Oosterlaar [75] demonstrated that five days of CrM loading (20 g/day), followed by six weeks of maintenance dosing (2 g/day), elevated muscle CR concentrations and sustained peak power improvements during repeated 12-second cycling sprints, despite partial regression of CR stores toward baseline. Similarly, long-term of CrM supplementation increased fat-free mass, total bench press volume, and cycling work output, highlighting CR’s potential for long-term adaptations [76]. Even shorter CrM protocols yield substantial benefits, while a loading dose of 20 g/day increased peak cycling power by 4% during six consecutive ten-second sprints [77]. Additionally, while another study demonstrated that acute CR supplementation enhanced anaerobic running capacity during a three-minute high-intensity bout [78].

Autonomic effects of acute CrM supplementation were observed in HRV improvements during the first session, with significant increases in RMSSD (*d* = 2.99) and HF (*d* = 2.76), reflecting enhanced parasympathetic reactivation [79]. This aligns with recent research reporting that CrM facilitates faster HRV recovery post-resistance exercise, due to its ability to mitigate oxidative stress [70]. Another recent study demonstrated that CR reduces lipid peroxidation and stabilises autonomic function [72]. However, HRV benefits diminished in the second session, reinforcing the hypothesis that accumulated fatigue may overshadow CR’s autonomic advantages during repeated high-intensity efforts [36].

### 4.2. Recovery effects of CrM supplementation

Neuromuscular recovery was superior with CrM supplementation, as sustained CMJ performance at 24 h (*d* = 1.10) and 48 h (*d* = 1.26) reflected preserved explosive strength. CrM attenuates post-exercise jump height declines by preserving ATP availability [80]. However, the lack of SJ improvements contrasts with CMJ results, potentially due to SJ’s reliance on concentric strength, which is less sensitive to CR’s effects on stretch-shortening efficiency [81]. Mechanistically, CR enhances titin phosphorylation, improving eccentric force absorption during CMJ [82].

CrM accelerates the restoration of PCr stores, which is critical for rapid ATP regeneration during repeated bouts of exercise. Supplementation of 20 g/day of CrM for 5 days elevates intramuscular total CR by 50% (20–40% as PCr), establishing a biochemical basis for its recovery benefits [5]. This PCr replenishment was directly linked to performance outcomes [18], where CrM supplementation (20 g/day for 5 days) increased peak torque production during repeated maximal contractions, particularly in later exercise bouts (bouts 2–4), compared to placebo. These findings align with Casey, Constantin-Teodosiu [69], who showed that 20 g/day CrM for 5 days reduced ATP loss by 30% during repeated 30-s cycling sprints, despite a 4% increase in total work output. By preserving ATP availability, CrM mitigates the cumulative fatigue associated with high-intensity efforts, a mechanism corroborated by Cooke, Rybalka [80], who linked CrM to sustained CMJ performance at 24 h (*d* = 1.10) and 48 h (*d* = 1.26) post-exercise.

Recent molecular insights reveal CR’s role in eccentric force absorption. CR enhances titin phosphorylation, a structural protein critical for passive muscle stiffness, thereby improving energy dissipation during stretch-shortening cycles (e.g. CMJ) [82]. This explains why CR sustains explosive strength recovery but shows limited effects on concentric-dominant tasks, which rely less on eccentric efficiency [81].

Perceptually, CrM supplementation reduced DOMS in upper (*d* = 1.15) and lower limbs (*d* = 1.04), consistent with its anti-inflammatory and cell-volumizing properties. CrM lowers creatine kinase (CK) post-exercise by stabilising sarcolemma integrity [83]. Furthermore, CrM demonstrated the capacity to minimise DOMS, particularly in workouts with high eccentric loads (e.g. BS), however benefits are more obvious in the lower limbs due to glycolytic demand [24,84]. CR attenuates exercise-induced muscle damage by stabilising sarcolemma integrity, where Santos, Bassit [83] found that CrM supplementation lowers post-exercise CK levels by 15–20%, reflecting reduced sarcolemma permeability. This aligns with Fukuda, Smith [78], who reported that acute CrM supplementation (20 g/day for 5 days) enhanced anaerobic running capacity during a 3-minute high-intensity bout, likely by minimising membrane disruption and subsequent inflammatory cascades. Doma, Ramachandran [85] confirmed CR’s efficacy in reducing DOMS particularly in exercises with high eccentric loads. These anti-inflammatory effects are partly mediated by CR’s osmotic properties, which increase muscle water content and reduce oxidative stress by scavenging reactive oxygen species [74].

### 4.3. Limitations

Our trial was designed to evaluate acute and short-term effects; conclusions about long-term adaptations fall outside the study’s scope. Given our study findings, several limitations should be acknowledged. Extensive randomised controlled trials and consensus statements already document long-term safety and efficacy of CrM; our results therefore complement, rather than duplicate, this literature by focusing on acute/short-term responses in resistance-trained males [4,22,86–88]. Although the study was a priori powered for our within-subject primary endpoint, the homogeneous sample (young, recreationally resistance-trained males) limits external validity. Future work should test broader demographics and potential moderators (training status, baseline CR stores, sex). Third, because testing sessions were separated by seven days, complete normalisation of intramuscular creatine cannot be fully ruled out. Although this duration likely minimised acute carryover, as supported by comparable baseline HRV, performance, and lactate measures across sessions, full return to baseline typically requires approximately four to six weeks after supplementation cessation [4,24]. Nevertheless, the present shorter interval aligns with recent applied crossover investigations that also employed seven-day washout periods [40,41], which have demonstrated this duration to be sufficient to limit acute residual effects without disrupting ongoing training. Our decision thus represents a deliberate balance between methodological control and ecological validity, reflecting real-world athletic practice where extended washout periods are often impractical during continuous training or pre-competitive phases. Fourth, only males were enroled to avoid menstrual-cycle–related variability in HRV and performance; cycle-phase control would have required additional sessions beyond this pilot. Future studies are encouraged to include females with appropriate cycle-phase controls. Additionally, while strength performance and HRV were assessed, biochemical markers of muscle damage and oxidative stress were not. Future studies should integrate these measures for a more comprehensive analysis. Lastly, we minimised dietary confounding by instructing participants to avoid creatine-rich foods and all supplements for 72 h before each visit and throughout testing; however, we did not collect detailed diet logs or biomarkers of intake. Future trials will use standardised menus or dietary logs and, where feasible, biomarkers to verify adherence.

## 5. Conclusion

A 3-day CrM supplementation protocol (initial full dose pre-training) enhanced strength performance and neuromuscular recovery in resistance-trained males, improving repetitions, peak velocity, and power during BP/BS at 60–80% 1RM. CrM also reduced post-session heart rate, muscle soreness (24–48h), and preserved CMJ performance while improving HRV. This strategy may benefit athletes undertaking multiple weekly sessions. Future studies should investigate longer-term effects, sex-specific responses, and biochemical markers to clarify CrM’s role in training adaptation and recovery.

## Data Availability

The data included in this study are available upon reasonable request.
